# Oral Presentations 7

**DOI:** 10.1038/sj.bjc.6601931

**Published:** 2004-06-22


### ORAL PRESENTATIONS 7

### 7.1

### TARGETING SMRT CO-REPRESSOR COMPLEXES AS A NOVEL CHEMOPREVENTION STRATEGY FOR PROSTATE CANCER

L.M. Gommersall [4], F.L. Khanim [1], D.M. Peehl [2], A.P. Doherty [4], N.D. James [3], M.J. Campbell [1]
[1] Institute of Biomedical Research, University of Birmingham, Birmingham, United Kingdom, [2] Department of Urology, Stanford University, Stanford, United States, [3] Division of Cancer Studies, University of Birmingham, Birmingham, United Kingdom, [4] Department of Urology, Queen Elizabeth Hospital, Birmingham, United Kingdom

We hypothesized that epigenetic mechanisms silence the responsiveness of key antiproliferative target genes for the vitamin D receptor resulting in insensitivity. Supportively using quantitative real time RT-PCR (Q-RT-RCR) we found elevated mRNA levels of the co-repressor SMRT in 1α,25(OH)$_2$D$_3$-resistant prostate cancer cell lines. Similarly 10/15 primary tumour cultures (including 3 matched to normal cells from the same donors) had elevated SMRT.
Targeted strategies towards SMRT complexes with combinations of 1α,25(OH)$_2$D$_3$ and the histone deacetylase (HDAC) inhibitor Trichostatin A (TSA) resulted in synergistic inhibition of proliferation, and co-operative upregulation of genes including MAPK-APK2 and GADD45α. MRNA time course studies measured by Q-RT-PCR combined with protein and inhibitor studies confirmed these patterns and significance of regulation. Supportively primary cancer cultures with elevated SMRT demonstrated suppressed GADD45α induction compared to matched normal controls, and siRNA towards SMRT in PC-3 cells significantly enhanced GADD45α induction.
These actions are not restricted to the VDR as multiple nuclear receptor ligands synergise with the clinically relevant HDAC inhibitor suberoylanilide hydroxamic acid (SAHA). High dose SAHA alone (≥ 2 μM) disrupted cytokiniesis and induced apoptosis, whereas doses ≤ 1 μM resulted in a G$_1$ arrest associated with a rapid induction of p21$^{(waf1/cip1)}$ mRNA and protein. We subsequently pursued the combinatorial antiproliferative action of SAHA (0.5 μM) with bezofibrate (BF) (which targets PPARα and γ), and demonstrated synergistic antiproliferative actions concomitant with cell cycle arrest. Similarly siRNA towards SMRT resulted in enhanced upregulation of GADD45α in response to BF. These data are consistent with a model of SMRT-mediated promoter-specific epigenetic silencing of multiple NR target genes and highlights a novel chemotherapy target.

### 7.2

### FIBROBLAST GROWTH FACTOR (FGF) 17 EXPRESSION IS UP-REGULATED HUMAN PROSTATE CANCER AND CORRELATES TO CLINICAL PROGNOSIS

R Heer , D Douglas , CN Robson , HY Leung
Urology Research Group, Northern institute for cancer research, Newcastle upon Tyne, United Kingdom

**Introduction:** Over-expression of fibroblast growth factor-8 (FGF8) in human prostate cancer is associated with clinically aggressive disease. Among different members of the FGF family, FGF17 and FGF8 share a high sequence homology and have similar patterns of expression during embryogenesis. We tested the clinical significance of FGF17 expression and its *in vitro* function in prostate cancer cells.
**Methods:** We studied forty resected prostate specimens, using semi-quantitative RT-PCR, from patients with benign prostatic hyperplasia (BPH, n=12) and prostate cancer (CaP, n=28; Gleason sum scores 3 - 10). In addition, 85 CaP (Gleason sum scores 5 – 9) were examined using immunohistochemistry with an optimised protocol for FGF17 immunoreactivity and findings were correlated with clinical parameters.
**Results:** Semi-quantative RT-PCR analysis for mRNA expression demonstrated a 4 fold up-regulation of FGF17 expression in high grade CaP (Gleason sum score 7 – 10) specimens when compared to BPH ($p < 0.0001$). Both immunohistochemistry and RT-PCR studies revealed a significant linear correlation with increasing Gleason sum score of cancer and FGF17 mRNA and protein expression ($p<0.0001$, Rho=0.99). Survival analysis showed that men with tumours displaying high levels of FGF17 expression had a worse prognosis ($p = 0.044$) and was associated with the presence of metastases ($p <0.0001$). Furthermore, we demonstrated FGF8 mediated induction of FGF17 in prostate cancer cell lines (LNCaP, DU145 and PC3M). This was further validated using a neutralising antibody specific to FGF8 which resulted in down-regulation of FGF17 expression.
**Conclusions:** Our data support a role for FGF17 in human prostate carcinogenesis and has revealed evidence for the control of FGF17 expression by FGF8.

### 7.3

### PREDICTION OF SKELETAL COMPLICATIONS IN METASTATIC BONE DISEASE AND STRATEGIES FOR MANAGEMENT OF TREATMENT-INDUCED BONE LOSS

J E Brown
University of Sheffield, Weston Park Hospital, Sheffield, United Kingdom

The skeleton is the most frequent site of metastasis from a range of common cancers and bone metastasis results in skeletal complications which can be devastating to quality of life. At the other end of the spectrum, combination chemotherapy and hormone therapy may be curative, but may lead to long term bone loss and skeletal morbidity. Bisphosphonates have made a major contribution to the management of these conditions, but there is a need to develop methods for prediction of risk of skeletal complications and assessment of response to therapy, in order to direct treatment to obtain optimum benefit.
In 121 patients with metastatic bone disease, the bone resorption marker N-telopeptide (NTX) was measured at monthly intervals and skeletal complications were recorded. There was a strong correlation between skeletal complications and NTX levels ($r = 0.62$, $p<.001$) and NTX was highly predictive for the skeletal complications experienced by a patient in the following 3 months. This was the first study to demonstrate such a relationship and was the foundation for a further larger study in which we used data from 3,000 patients in international, multicentre trials with zoledronic acid. The larger study confirmed that bone markers are powerful tools in predicting impending skeletal morbidity. Additionally, in the first study of its kind, we demonstrated the value of bone markers in tailoring dosing and scheduling of a bisphosphonate to individual patients.
In a trial of 216 long term survivors from testicular cancer and lymphoma, we have shown (reassuringly) that there is no significant effect of chemotherapy on bone loss compared with a control group. In a further trial in patients with treatment-induced osteoporosis or osteopenia, the feasibility of single annual doses of zoledronic acid to treat bone loss is being assessed (to date n = 74). Initial results show that this is feasible, convenient and effective with sustained increases in bone mineral density ranging from 2.8% to 3.7% from a single drug infusion.

### 7.4

### ACCURACY OF CT SCAN IN STAGING OF RENAL CELL CARCINOMA

BK Somani , A Chakravarti , MA Jones , K Kadow
Sandwell & West Birmingham hospitals NHS Trust, Birmingham, United Kingdom

To evaluate the efficacy of CT scan in the pre-operative staging of Renal tumours.
In a retrospective cohort study of 45 patients who had radical nephrectomy for renal tumour between 1995 – 2000, CT scan and histopathological findings were compared. We looked at the tumour size, capsular invasion, renal vein invasion and lymph node status on CT scan and histopathological reports.
CT scan was accurate in predicting the size of tumour in 14% of cases. The mean variation was 1.6 cm (Range : 0.5 – 8 cm). Capsular invasion was detected by CT scan in 45% (6 of 14) patients who had true capsular invasion on histopathology. Renal vein invasion was not identified by CT scan in any of the 6 patients, who had histopathologically proven invasion. Lymph Node invasion was identified in 3 patients on CT scan of which only 1 was a true invasion seen on histology. However it failed to identify positive lymph nodes in 2 other patients who had histologically proven lymph node metastasis.
Although CT scan is a useful tool in the diagnosis of renal masses, its accuracy in staging renal tumours preoperatively is limited.

© 2004 Cancer Research UK

### 7.5

### PRELIMINARY EXPERIENCE USING HIGH-INTENSITY FOCUSED ULTRASOUND FOR THE TREATMENT OF KIDNEY AND LIVER TUMOURS

RO Illing [1], JE Kennedy [1], F Wu [2], GR terHaar [3], RR Phillips [1], AS Protheroe [1], MR Middleton [1], DW Cranston [1]
[1] Churchill Hospital, Oxford, United Kingdom, [2] Clinical Center for Tumor Therapy, Chongqing University of medical Sciences, Chongqing, China, [3] Royal Marsden Hospital, Sutton, United Kingdom

High-intensity focused ultrasound (HIFU) provides a potential non-invasive alternative to conventional therapies. The extracorporeal ultrasound-guided Model-JC Tumor Therapy System (HAIFU™ Technology Company, China) has been used to evaluate the safety and feasibility of treating renal and liver tumours. A part of each tumour was treated under general anaesthesia in a single session according to 3 trial protocols. MRI after 12 days provides an initial assessment of response. In both the 1st and 2nd protocols, patients with liver tumours are treated and then imaged on day 12. Those in the 1st protocol are followed up with further MRI evaluation at 3 months. In the 2nd protocol the patients undergo partial hepatectomy enabling histological evaluation of the ablated region. The 3rd protocol evaluates the treatment of large kidney tumours prior to elective nephrectomy. 16 liver and 5 kidney patients have been treated to date. Of the 16 liver patients, 10 have been enrolled into the first protocol, one non-evaluable. One patient died prior to post-treatment MRI (unrelated to treatment). 7 of the remaining 8 patients have shown clear evidence of tumour ablation on post-treatment MRI. 6 liver patients and 5 kidney patients have gone on to surgery. Post-HIFU MRI revealed volumes of ablation in all 6 liver patients which correlated with the histological findings. Post-treatment MRI evaluation of the kidney tumours showed evidence of ablation in 2 of the 5 cases, however there was only clear histological correlation in one of these. Mild transient pain was reported by 55% of patients, and more severe pain in one case. Superficial skin burns were seen in 7 patients (35% of cases). Our early experience suggests that HIFU treatment of liver and kidney tumours is safe and has few complications or side effects. MRI evidence of ablation has been apparent in 80% of those patients treated (liver 93%, kidney 40%), and histological changes consistent with ablation have been recorded in 64% of patients (liver 100%, kidney 20%).

### 7.6

### INFLIXIMAB: A PHASE II TRIAL OF THE TUMOUR NECROSIS FACTOR (TNFα) MONOCLONAL ANTIBODY IN PATIENTS WITH ADVANCED RENAL CELL CANCER (RCC)

NR Maisey , K Hall , C Lee , E Timotheadou , R Ahern , T Eisen , M Gore
Royal Marsden Hospital, London, United Kingdom

**Background:** Several small peptides are over-expressed in RCC including TNFα. Trials of thalidomide have shown promising results in advanced RCC, possibly due to the down-regulation of some of these peptides including TNFα. Infliximab is a chimeric human / mouse monoclonal antibody against TNFα. We have performed a phase II study of this in pts with RCC.
**Methods:**This was an open, single arm, phase II study using a 2 stage Gehan design. Eligibility criteria: metastatic RCC progressing after immunotherapy (IFα +/or IL2), performance status (PS) 0-2, life expectancy >12 weeks. Infliximab (5mg/kg) was administered on weeks 0, 2, 6, 14, 22, 30. Treatment could continue until progressive disease (PD). Response was assessed before each dose from week 6.
**Results:** 19 patients are evaluable for toxicity and 18 for response. Median follow-up: 125 days (range 25-318). Median age: 53 (35-76). First-line immunotherapy included: IFα (n=12); IFα/IL2/5-Fluorouracil (n=7). Additional lines of treatment prior to study entry were given to 8 patients including IF?/IL2/5-Fluorouracil, GW57016, Vinflunine, B-Raf inhibitor, gemcitabine/capecitabine, medroxyprogesterone acetate. Patients received the following number of doses: 1 (n=19); 2 (n=18); 3 (n=12); 4 (n=5); 5 (n=3); 6 (n=1). 2 patients achieved partial response (PR) at 14 and 30 weeks. 1 late response (15 weeks) was observed following initial PD at 6 weeks. 4 patients have ongoing disease control (PR + stable disease) at 1,2,3,9 months. Probability of survival at 9 months is 47% (95% CI 13-75). Median progression free survival is 56 days (95% CI 31-81). Other than 1 episode of grade 3 hypersensitivity there were no significant toxicities.
**Conclusions:**This study shows that infliximab is non-toxic and active in RCC. 2 patients achieved PR and 1 patient had a late response. Further studies are warranted to investigate the potential role of infliximab in the management of RCC.

### 7.7

### A RANDOMISED TRIAL OF CARBOPLATIN VERSUS RADIOTHERAPY FOR STAGE I SEMINOMA OF THE TESTIS, FOLLOWING ORCHIDECTOMY: MRC TE19/EORTC 30982

RTD Oliver [1], M Mason [2], H Von der Maase [3], SP Stenning [4], SJ Kirk [4], GJS Rustin [5], GM Mead [6], R de Wit [7]
[1] St Bartholomew's Hospital, London, United Kingdom, [2] Velindre Hospital, Cardiff, United Kingdom, [3] Aarhus University Hospital, Aarhus, Denmark, [4] MRC Clinical Trials Unit, London, United Kingdom, [5] Mt Vernon Hospital, Northwood, United Kingdom, [6] Southampton General Hospital, Southampton, United Kingdom, [7] Erasmus Medical Centre, Rotterdam, Netherlands

**Background:** Prompted by evidence that radiotherapy (R) for stage I seminoma increases late malignant and cardio-vascular events, MRC trials have investigated reduced radiation volume (TE10) and reduced dose(TE18) without loss of effectiveness. The latest trial compares radiation and 1 course of carboplatin (C) and the initial results of this study are presented
**Methods:** Randomisation was between R and 1 course of C AUCx7. An optional randomisation was allowed between 20 Gy/10f and 30 Gy/15f as in TE18. The trial was powered to exclude absolute differences in the 2 year relapse rates of >3%.
**Results:** From June 96 to March 01 1447 patients (pts) were randomised (ratio 3: 5) between C and R. Of pts randomised to R 13% had dogleg field and 87% PA strip. Median follow up (FU) is now 3 years and over 90% of patients have at least 2 years FU. Relapse-free survival rates for R vs C (95%CI) are 97.2% (95.9, 98.1) v 98.1% (96.6, 98.9) at 2 years and 96.6% (95.2, 97.6) v 95.4% (93.3, 96.9) at 3 years; HR 1.39 (90% CI: 0.92, 2.11) p=0.195; the 90% CI excludes an increase in relapse rates in the C arm of more than 3% at 2 years and of more than 4% at 3 years. Pattern of relapse varied, sites being the PA nodes only in 70% (C) v 7% (R), and pelvis relapse in 4% (C) vs 28%(R). Second germ cell tumours (GCTs) have been reported in 1 pt allocated C and 7 allocated R, non-GCTs 2 C vs 4 R. No disease or treatment related deaths have been reported after C and only 1 after R; non-cancer deaths 1 (C) vs 2 (R). There was no difference in gonadal function(FSH, LH & Testosterone) between arms at 1 & 2 years
**Conclusions:** With a median FU of 3 years, an absolute increase in relapse rate in the C arm of more than 3% at 2 years can be excluded reliably, early data on new primary cancers favour the C group and to date there have been no disease or treatment related deaths. Further FU is needed to confirm that these results are maintained beyond 3 years.

© 2004 Cancer Research UK

